# Emerging Viral Infections in Pregnancy: What’s New in 2022?

**DOI:** 10.3390/v15040889

**Published:** 2023-03-30

**Authors:** Léo Pomar, David Baud

**Affiliations:** 1 Department Woman-Mother-Child, Lausanne University Hospital, University of Lausanne, 1011 Lausanne, Switzerland; 2 School of Health Sciences (HESAV), University of Applied Sciences and Arts Western Switzerland, 1011 Lausanne, Switzerland

Dear contributors and readers,

In this 2022 edition of the Special Issue of *Viruses* entitled “Emerging Virus Infections in Adverse Pregnancy Outcomes”, we have received and published some very relevant studies on these topics.

We have received several high-quality studies on SARS-COV-2 infections during pregnancy, presenting placental pathology after exposure to SARS-COV-2, some maternal comorbidities affecting the immune response to SARS-COV-2, and biological markers of severe COVID-19 in pregnant women. Concerning congenital Zika infections, special attention has been paid to late manifestations in children with congenital Zika syndrome, including studies reporting urinary, neurologic, neurosensory, and feeding impairments among them. The challenge of this new Special Issue was also to extend its scope to other emerging or endemic infections that remain understudied. Therefore, we have published a review summarizing the acute and chronic complications of influenza virus infection during pregnancy, a review summarizing the latest knowledge and guidelines on mpox in pregnancy, and a very rare case of congenital lymphocytic choriomeningitis diagnosed in utero. However, a key additional focus of this Special Issue was on cytomegalovirus, with the publication of a comprehensive review on CMV prevention in pregnancy, including advances in vaccines and antiviral therapies in utero; and the publication of two studies on specific serological and molecular markers of primary and non-primary CMV infection in pregnancy, emphasizing the growing interest in non-primary infections that remain difficult to diagnose with commonly used tools. 

We would like to express our thanks to all the researchers and clinicians who contributed to this Special Issue, and we hope to work with them again in the future. 

